# Comparative transcriptomics in the Triticeae

**DOI:** 10.1186/1471-2164-10-285

**Published:** 2009-06-29

**Authors:** Andreas W Schreiber, Tim Sutton, Rico A Caldo, Elena Kalashyan, Ben Lovell, Gwenda Mayo, Gary J Muehlbauer, Arnis Druka, Robbie Waugh, Roger P Wise, Peter Langridge, Ute Baumann

**Affiliations:** 1 Australian Centre for Plant Functional Genomics, Univ of Adelaide, PMB 1 Glen Osmond, SA 5064, Australia; 2 Dept. of Plant Pathology and Center for Plant Responses to Environmental Stresses, Iowa State Univ., Ames, IA 50011-1020, USA; 3 Dept. of Agronomy and Plant Genetics, Univ of Minnesota, St. Paul, MN 55108, USA; 4 Scottish Crop Research Institute, Invergowrie, Dundee DD2 5DA, UK; 5 Corn Insects and Crop Genetics Research, USDA-ARS, Iowa State Univ, Ames, IA 50011-1020, USA

## Abstract

**Background:**

Barley and particularly wheat are two grass species of immense agricultural importance. In spite of polyploidization events within the latter, studies have shown that genotypically and phenotypically these species are very closely related and, indeed, fertile hybrids can be created by interbreeding. The advent of two genome-scale Affymetrix GeneChips now allows studies of the comparison of their transcriptomes.

**Results:**

We have used the Wheat GeneChip to create a "gene expression atlas" for the wheat transcriptome (cv. Chinese Spring). For this, we chose mRNA from a range of tissues and developmental stages closely mirroring a comparable study carried out for barley (cv. Morex) using the Barley1 GeneChip. This, together with large-scale clustering of the probesets from the two GeneChips into "homologous groups", has allowed us to perform a genomic-scale comparative study of expression patterns in these two species. We explore the influence of the polyploidy of wheat on the results obtained with the Wheat GeneChip and quantify the correlation between conservation in gene sequence and gene expression in wheat and barley. In addition, we show how the conservation of expression patterns can be used to elucidate, probeset by probeset, the reliability of the Wheat GeneChip.

**Conclusion:**

While there are many differences in expression on the level of individual genes and tissues, we demonstrate that the wheat and barley transcriptomes appear highly correlated. This finding is significant not only because given small evolutionary distance between the two species it is widely expected, but also because it demonstrates that it is possible to use the two GeneChips for comparative studies. This is the case even though their probeset composition reflects rather different design principles as well as, of course, the present incomplete knowledge of the gene content of the two species. We also show that, in general, the Wheat GeneChip is not able to distinguish contributions from individual homoeologs. Furthermore, the comparison between the two species leads us to conclude that the conservation of both gene sequence as well as gene expression is positively correlated with absolute expression levels, presumably reflecting increased selection pressure on genes coding for proteins present at high levels. In addition, the results indicate the presence of a correlation between sequence and expression conservation within the Triticeae.

## Background

Considerable divergence has occurred between bread wheat (*Triticum aestivum*) and barley (*Hordeum vulgare*) since evolution from a common ancestor 10–14 million years ago. Since then, these two members of the *Triticeae *have been subjected to largely parallel processes of cultivation and domestication, starting in the fertile crescent over 10,000 years ago [1]. Barley has remained diploid with a base chromosome number of 7 (HH genome, 2n = 2x = 14) while bread wheat is the product of a series of hybridization events between related species that has resulted in an allo-hexaploid genome with three homoeologous sets of 7 chromosome pairs (AABBDD genome, 2n = 6x = 42 [2]). Despite these major genomic perturbations during its evolution, genetic mapping [3] and detailed structural genomic studies [4] have shown that the wheat and barley genomes are highly conserved. Indeed, barley chromosomes can even be substituted for wheat chromosomes [5]. As a consequence of its simplified genetics, many have suggested that barley is a good genetic model for its genetically more complex cousin. This assertion is supported by the broad range of common morphological and developmental characteristics shared by both species, though fundamental biological differences do exist (such as spike and spikelet morphology).

Polyploidization is common across the plant kingdom and the process has been associated with a range of changes in newly synthesized hybrids of several species. These include the genome-wide removal of some (but not all) duplicated, and hence redundant, genetic information, sub- and/or neo-functionalization of duplicated genes, pseudogenization, differential cytosine methylation and epigenetic reprogramming of gene expression (silencing and activation), and transposable element activation (reviewed in [6]). Levy and Feldman [7] summarized some of the major consequences resulting from the recent polyploidization of the wheat genome. In common with other plant species, the outcome for wheat was more than simply the additive combination of genomes and included many of the features described across the species range [8-10].

Wheat is an important species for studying the impact of polyploidization because it is a relatively recent polyploid. Moreover, the outcomes can be studied in very early generations because it is possible to artificially re-synthesize polyploids from their diploid and tetraploid relatives. In such cases, epigenetic silencing of duplicated genes appears to be a common response, with indications of reciprocal silencing in different organs an early sign of sub-functionalization [11-15]. Gene activation or silencing may also occur as a result of transcriptional interference associated with stochastic rearrangements of non-coding RNA [8]. Over longer time frames, the evolutionary consequences of such events are better observed in ancient polyploids. In Arabidopsis (an ancient tetraploid), for example, Blanc and Wolfe [16] reported that more than half of the observed gene pairs retained in the genome exhibited differential transcript abundance in different tissues. An immediate impact of polyploidy is therefore to provide the raw genetic material for adaptation and the evolution of phenotype.

The close evolutionary relationship between wheat and barley, reflected in largely parallel morphological and developmental patterns, makes a comparison of their transcriptomes particularly intriguing. It may provide insight, for example, into consequences of speciation and polyploidization. Ideally a genomic-scale comparison of this sort would be carried out once the genomes have been sequenced. This would permit the reliable disentanglement of the evolutionary relationships between individual genes and also provide the foundation on which to build dependable expression analysis platforms. Regrettably, the size and complexity of the wheat and barley genomes has been a major impediment to full-scale sequencing, so that even the diploid barley genome is not expected to be available before 2012 . In short, among plants comparative transcriptomics is rare: comprehensive pair-wise comparisons have so far only been carried out in rice and Arabidopsis [17], various cotton species [18] and in poplar and Arabidopsis [19]. Recently, a three-way study between Arabidopsis, poplar and rice has also appeared [20].

Compared to genome-wide studies, comparisons of expression patterns of individual orthologous gene pairs, individual gene families and/or in connection with a particular phenotypic characteristic are more frequent. For example, Mangelsen et al. [21] compared, within a number of tissues, expression patterns of members of the WRKY transcription factor family among barley, rice and Arabidopsis and found that, at least within this gene family, coordinated conservation of expression patterns and sequence. Horvath et al. [22] found that groups of genes associated with cell division were consistently expressed preferentially in shoot apices in Arabidopsis, wild oats, poplar and leafy spurge. Differential gene expression, on the other hand, has been observed in some members of the ZIP and NAS metal homeostatis gene families in two closely related Arabidopsis species when exposed to both low and high Zn levels, presumably associated with different Zn accumulation patterns in these two species [23]. Analogously, differential time-dependent expression of a small number genes in response to salt stress in both barley (relatively salt tolerant) and rice (relatively salt sensitive) were studied by Ueda et al. [24], while Taji et al. [25] performed a similar comparative study in salt cress (tolerant) and Arabidopsis (intolerant).

Comprehensive Affymetrix GeneChip platforms have now been developed for both wheat and barley, based on extensive EST collections for both species (Ref. [26]; ). The Barley1 GeneChip has already been used to develop an atlas of gene expression covering the entire developmental cycle of the barley cultivar Morex [27] and intra-species varietal comparisons have been carried out both for Morex and Golden Promise [27,28] as well as Morex and Steptoe [29,30]. Taking advantage of these resources, we have sampled a similar set of biological material collected through the developmental cycle of wheat (Chinese Spring), grown under near-identical conditions to those in Druka et al. [27]. This permits the first comprehensive comparison of developmental expression patterns in these two important crop species. We report on this transcriptome-wide comparison here. At the same time, in order to facilitate more detailed studies of individual homologous genes motivated, say, by particular phenotypic differences as in [21-25], we make available a convenient web-based comparative tool enabling access to the developmental expression profiles of *any *individual wheat and barley homologs probed by the two GeneChips.

It is well known that meaningful comparative expression analyses using microarray platforms based solely on EST collections can be difficult because of the frequent and confounding presence of multiple splice forms, paralogs and orthologs, as well as, in the case of polyploids, homoeologs with near-identical sequence [31]. Because of this, we have also investigated, in some detail, the specificity of the Wheat GeneChip to individual homoeologs and expended considerable effort to avoid misidentification of orthologs in the two species.

## Results

Gene expression measurements were carried out on a developmental tissue series for wild-type wheat (cv. Chinese Spring) using the Affymetrix Wheat GeneChip. Tissues and developmental stages were chosen to match the barley (cv. Morex) tissue series of Druka et al. [27], employing the Barley1 GeneChip, as closely as possible. They consisted of root tissue at two different developmental stages, leaf, crown, caryopsis, anther, pistil, inflorescence, bracts, mesocotyl, endosperm, embryo and coleoptile (for details, see Materials and Methods: Microarray experiment). This wheat expression dataset may be obtained from and visualized at PLEXdb , experiment TA3, or GEO , Experiment GSE12508. Because the 61,115 probesets on the Wheat GeneChip reflect the complete collection of publically available wheat ESTs at the time of design of the chip, this dataset serves as an 'expression atlas' for hexaploid wheat. While in this paper we concentrate on the comparison of the barley and wheat transcriptomes, the comprehensive nature of the dataset means that it may be used by wheat researchers seeking to explore correlations in transcript levels across tissues to discover putatively co-regulated and/or functionally related genes and it provides a baseline against which transcription in biotically or abiotically stressed plants, other cultivars and mutants may be compared.

Using single varieties as representatives for both barley and wheat may to some extent be an oversimplification as it is known that considerable variation can exist among varieties of the same species. Indeed, extensive intra-species variation in barley has been used (employing the Barley1 GeneChip) as a genotyping tool for a cross between the barley varieties Steptoe and Morex [29,32] and for expression polymorphisms among Morex, Steptoe, OWB REC, OWB DOM, Barke, Haruna Nijo, Golden Promise and Optic (PLEXdb accession BB20, ArrayExpress E-TABM-113). We compared the relative importance of intra- and interspecies variation by making use of a common series of 6 tissues from the barley cultivars Golden Promise and Morex (taken from Ref. [27]).

For the purpose of the comparison of the transcriptomes of the two species, we found it convenient to define a set of probesets, obtained by eliminating those probesets that were potentially unreliable for various reasons (for details, see Table 1 and Materials and Methods: The Wheat GeneChip). This set of probesets will be referred to as the "high quality" set throughout this paper. This resulted in an expression dataset for 13,822 wheat probesets that could be compared, across 13 tissues, with the equivalent dataset obtained with 12,549 barley probesets hybridized by Druka et al. [27].

**Table 1:** Summary of the types of probesets present on the Affymetrix Wheat GeneChip.

**Probeset type^a^**	**Number of probesets**
Total number of probesets	61115
Cross-hybridizing (_s_at,_x_at,_a_at)	12704
Ambiguous orientation (A1)	10643
Members of 5' (i.e. "prune") set	32578
"High quality" probesets	13822

^a^The total number of probesets does not include reporters and controls. Note that there is some overlap among the various probeset groups in this table.

### Polyploidy and the Wheat GeneChip

A comparison of the transcriptomes of barley and wheat is complicated by the fact that the latter is a hexaploid, but the design of the probesets on the wheat GeneChip did not specifically take this polyploidy into account. Hence, depending on the stringency of the probeset design, an expression profile obtained from the wheat GeneChip may receive contributions from one, two or three homoeologs. This has the potential to complicate a comparative study of gene expression in the two species. We explored this issue by comparing the probes on the wheat GeneChip with known sequences of wheat homoeologs. In Mochida et al. [33], ESTs for 90 genes were assigned to the A, B and D genomes using nullisomic lines of Chinese Spring. These authors found that 11 genes had one of the homoeologs silenced while 79 exhibited transcript accumulation for all three homoeologs.

We extracted all 25-mer probe sequences on the wheat GeneChip that exhibited a perfect match to this set of 79 × 3 = 237 sequences. Discarding probes from those probesets which are known to either cross-hybridize or are likely to hybridize inefficiently (for details, see Materials and Methods: The Wheat GeneChip), as well as homoeologous triplets for which only a very small number (< 7) of matching probes are present on the GeneChip, left 56 homoeolog triplets for which relatively reliable expression information was available from the microarray.

**Figure 1:**
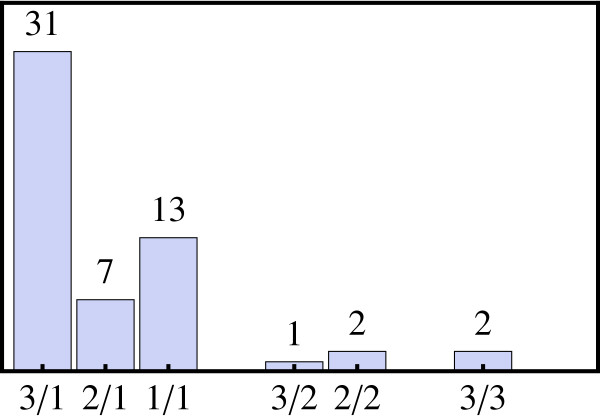
**Homoeologous sequences and the Wheat GeneChip**. The number of probesets with a significant (≥ 7) number of probes matching to 237 homoeologous sequences known to be expressed at some level is shown in this histogram. Categories are labelled as x/y, indicating that x out of 3 homoeologs are represented on the Wheat Genechip by y probesets. For example, "3/1" means that all (or almost all) probes from a single probeset match perfectly to all three homoeologs of a gene and hence that individual contributions from the homoeologs cannot be resolved; "3/2" indicates that the contribution from one homoeolog can be resolved, while the other two hybridize to a single probeset; similarly, "2/1" indicates that two homoeologs match probes from a single probeset, while the third homoeolog does not significantly hybridize to any probes. In only two cases, indicated by "3/3", can the expression of each of the three homoeologs be detected individually by probes from three separate probesets, while in the majority of cases homoeologous triplets (51 out of 56) are significantly matched by probes from only a single probeset.

The distribution of matching probes for these 56 triplets is shown in Figure 1. For more than half of the homoeologous triplets a significant number of probes (≥ 7, but in most cases 10 or 11) from a single probeset match perfectly to all three copies of the gene. For many of the remaining triplets either one or two homoeologs do not significantly hybridize to any probesets. As indicated by the three bars in the right half of Figure 1, only for five triplets can significant information be obtained from two or more probesets. At first sight, this lack of specificity might seem surprising, given that for these sequences the single nucleotide polymorphism (SNP) frequency across the A, B and D genomes is around 1 per 145 bases [33]. With this SNP frequency, one might have naively expected to find imperfect matches to any given homoeolog in roughly every sixth probe. It is likely that the observed specificity is lower than this because of the design of the wheat GeneChip, on which probes were specifically designed to the conserved regions of consensus sequences. In any case, by extrapolating from this rather small sample to the whole dataset we conclude that it is likely that for over ~90% of genes the measured expression profiles represent a sum of the individual profiles of the relevant homoeologs.

### Homolog identification on the Wheat and Barley1 GeneChips

Putative homologous sequences on the wheat and barley GeneChips were inferred using a clustering approach based on sequence similarity [34-38]. This methodology was chosen over, for example, direct putative ortholog identification via the traditional best reciprocal Blast hit (RBH) method [39-41] because (a) the latter is known to be unreliable in the presence of multiple paralogs [42], (b) the RBH method can lead to misidentifications due to the incomplete coverage of the wheat and barley genomes [43-45] and (c) the RBH method could not simultaneously detect the frequent presence of both 3' and 5' fragments of individual genes on the wheat GeneChip. For details relating to this clustering, see Materials and Methods: Homolog identification on the wheat and barley GeneChips.

As a resource for the Triticeae community, we have built an on-line tool where these "homologous clusters", as well as their associated expression profiles and sequence annotation, can be interrogated. This tool, the *WebComparator*, is available at . It may be used to assess similarities and differences of expression profiles of 10,708 individual homologous clusters. Expression profile differences may be due to biological differences, but quite frequently may simply reflect problems with hybridization efficiencies for individual probesets. The tool, therefore, is also useful in assessing the reliability of individual probesets on the Affymetrix Wheat and Barley1 GeneChips. Apart from similarities and differences in expression profiles of genes in individual homologous clusters, underlying global patterns are observable in the two datasets and it is those on which we focus here.

### Gene expression in wheat and barley is highly correlated

Direct gene-by-gene comparison of expression profiles in wheat and barley is complicated by the ambiguities associated with the many-to-many relationships characteristic of the homologous clusters. However, for the class of clusters consisting of exactly one wheat probeset and one barley probeset, ortholog association is less ambiguous: apart from the aforementioned general insensitivity to individual wheat homoeologues, one would expect this class to be enriched for probesets targeting single-copy and single-spliceform genes. Using only the "high quality" probesets (Table 1), this group of clusters consists of 1,875 sequence pairs.

**Figure 2:**
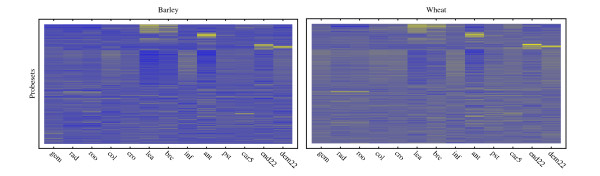
**Large-scale comparison of barley and wheat transcriptome**. These heat maps provide a comparative overview of gene expression for 1875 barley (left) and wheat (right) sequences. The same pattern of low (blue) and high (yellow) expression is clearly seen in both datasets. For this plot, expression profiles were centered and then sorted according to a hierarchical clustering of the barley data using single linkage and a correlation distance measure. Tissue abbreviations: gem – mesocotyl, rad – radicle, roo – root, col – coleoptile, cro – crown, lea – leaf, brc – bracts, inf – inflorescence, ant – anther, pst – pistil, car5 – caryopsis, end22 – endosperm, dem22 – embryo. Developmental time points are as described in [27] and include 2 day old embryos (gem, rad, col), 10 cm seedlings (roo, cro, lea), prior to anthesis (brc, inf, ant, pst) as well as 5 (car5) and 22 days (end22, dem22) after pollination. (see also Materials and Methods: Microarray Experiment).

**Figure 3:**
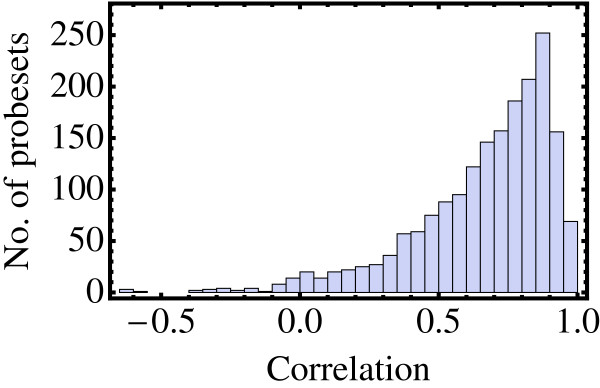
**Correlation of barley and wheat expression profiles**. The correlation distribution of 1,875 barley and wheat expression profiles is shown. Expression for putatively orthologous genes in barley and wheat tends to be highly correlated, with an average correlation of 0.66.

Transcript profiles for these probesets are shown as heat maps in Figure 2 where probesets have been sorted according to a hierarchical clustering for the barley dataset, defined via a single linkage correlation measure. The same pattern of gene expression is clearly visible in both the barley and wheat heat maps indicating that, by and large, gene expression in the two species is highly conserved. The same conclusion can be reached by inspection of the correlation distribution shown in Figure 3. The average correlation of the expression profile pairs in these 1,875 homologous clusters is 0.66.

### Systematic relative shifts in expression levels are frequent in wheat and barley

**Figure 4:**
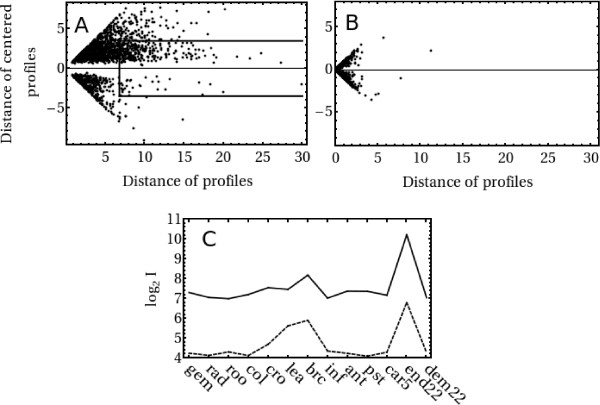
**Similarities and differences between wheat and barley profiles**. Panel A shows the Euclidean distance between wheat and barley expression profiles (x-axis) plotted against the Euclidean distance between their 'centered' profiles (y-axis) for the 1,875 "high quality" sequence pairs. In a centered profile, expression is measured relative to the average across tissues. Positive y-values correspond to average expression in barley greater than in wheat while negative y-values indicate average expression in wheat greater than barley. Expression profiles which have the same shape and overall magnitude of expression fall close to the origin of this plot, while expression profiles which have the same shape but are offset from each other simultaneously exhibit a small relative distance but large absolute distance (boxed region). For comparison, Panel B shows the analogous plot, on the same scale, for two barley cultivars Morex and Golden Promise (albeit over 6 rather than 13 tissues); data taken from Ref. [27]). Panel C shows a typical barley (solid line) and wheat (dashed line) expression profile identified from the boxed region in Panel A to be of similar shape but differing by an offset. In this case the offset corresponds to a signal intensity 8 times higher in barley than wheat. The particular example shown here corresponds to the wheat contig TaAffx.78909.1.S1_at and the barley Contig16549_at, annotated as a MYB transcription factor.

While expression profiles in wheat and barley are highly correlated, it is also of interest to know whether the *overall *level of expression is similar. In this case the Euclidean distance between corresponding expression profiles should be small. Indeed, the accumulation of points near the origin of the Euclidean distance plot shown in Figure 4A confirms this to be generally true. However, there are also a significant number of sequence pairs whose profiles are of similar shape but offset by significant amounts (boxed region in Figure 4A). An example of this, where expression in barley is roughly 8 times that in wheat, is shown in Figure 4C. The fact that there are many more points in the upper half of the boxed region in Figure 4A as compared to the lower half shows that if profiles are offset in this way, it is generally barley that exhibits a higher signal intensity than wheat. For comparison, Figure 4B presents an analogous plot to Figure 4A, involving the two barley cultivars Morex and Golden Promise (data taken from Ref. [27]). Comparison to Figure 4A shows that, on the whole, differences between these two barley varieties are negligible when compared to the differences between the two species.

**Figure 5:**
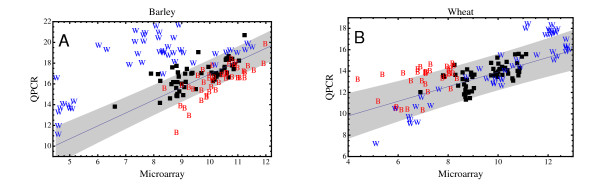
**Validation of selected barley and wheat expression profiles via QPCR**. A comparison of logarithmic fluorescence intensities, as measured on the microarrays, with logarithmic QPCR expression levels is shown. Barley data is shown in Panel A and wheat data in Panel B. Four wheat and four barley sequences where the average microarray fluorescence intensity for wheat is greater than barley give rise to the expression levels labelled by "W" (This comparison is carried out individually for each of the eleven tissues, hence there are a total of 44 points marked "W"). Three wheat and four barley sequences where the microarray fluorescence intensity for wheat is less than barley are marked by "B". Plain black data points indicate five sequence pairs where the microarray fluorescence intensities for wheat and barley are comparable. Data from these five sequence pairs were used to generate a calibration curve between the QPCR and microarray data in the form of a straight line fit (solid line) and associated 95% single prediction confidence limits (shaded region).

If this unexpected difference of signal intensities in wheat and barley were to reflect an underlying difference in mRNA levels for these genes it would be of interest to compare the corresponding protein levels in these two species. This might indicate a surprising shift of regulatory control from the transcriptional to the translational level. However, because overall shifts in signal intensities as measured by two different platforms can easily have a technical origin we have sought to independently verify the effect using quantitative real-time PCR (QPCR). The results are shown in Figure 5, where microarray signal intensities are plotted against expression levels as measured by QPCR for a number of different sequence pairs (for details, see Materials and Methods: Quantitative RT-PCR (QPCR) verifications). Four wheat and four barley sequences where the overall microarray fluorescence intensity in wheat is significantly higher than in barley are indicated by "W". Three wheat and four barley sequences where the microarray results for barley is significantly higher than wheat are marked by "B". Five sequence pairs where microarray fluorescence intensities are roughly the same for the two species are indicated as plain data points and were used to generate a calibration curve for the two types of measurements. The uncertainty of this calibration, in the form of single prediction 95% confidence limits, is indicated by shaded regions in Figure 5.

We first concentrate on those data points, marked by a "W", where the wheat profile obtained with the microarray is significantly higher than the corresponding barley profile. Expression data for barley (Panel A, Figure 5) show that these sequences exhibit a microarray fluorescence intensity that is significantly at odds with that expected from the corresponding QPCR signal. At the same time there is no sign of anomalous differences between the QPCR and microarray measurements for the corresponding wheat expression (Panel B, Figure 5). We conclude from this that for those orthologous pairs for which microarray measurements indicate expression in barley to be systematically lower than in wheat (i.e. the lower boxed portion in Figure 4A) the differences are likely to be due to a deficient signal obtained from the barley microarray, and not due to enhanced expression in wheat. We defer comment on the likely origin of this deficiency to the Discussion section.

For sequence pairs where the barley profile is systematically higher than its wheat counterpart (i.e. those shown in the upper boxed portion of Figure 4A and marked with a "B" in Figure 5) the situation is less clear. These data points do fall mostly within the calculated 95% confidence limits for a consistent QPCR and microarray signal and the data is, therefore, consistent with the conclusion that for a significant number of genes the transcriptional activity in wheat is indeed below that in barley. We caution, however, that at this stage one also cannot rule out that the discrepancy may be due to simultaneous small shifts of technical rather than biological origin, causing the barley fluorescence in the microarray data to be systematically increased and at the same time the wheat fluorescence to be systematically decreased somewhat from what they should be.

### 5' Probesets on the Wheat GeneChip hybridize unpredictably

The comparison of expression information between wheat and barley provides a unique opportunity to assess the reliability of the hybridization signal from the wheat GeneChip. This is important because the wheat microarray contains, apart from the usual probesets designed for sequences for which there is good evidence that they are near the 3' end of the gene (such as the presence of a poly-A tail), a large number of probesets for which this evidence does not exist (see Materials and Methods: The Wheat GeneChip; for convenience, we refer to the latter as "5' probesets"). Because of mRNA degradation away from the 3' end, one might expect the latter to lead to a reduced signal.

**Figure 6:**
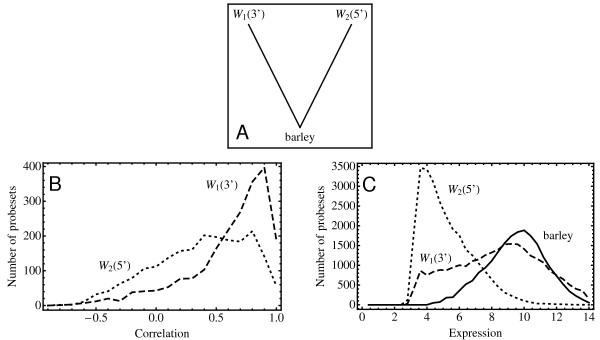
**Expression differences between wheat 3' and 5' probesets**. Comparison of expression profiles for wheat and barley allows an assessment of the reliability of the wheat 5' probesets. Homologous clusters of the type shown in Panel A suggest the simultaneous presence of both a 3' (W_1_) and 5' (W_2_) wheat probeset for the same gene that, at the same time, is homologous to a particular barley gene. The correlation of the expression profiles of these 3' and 5' probesets are shown in Panel B. On average, the profiles obtained from the 5' probesets show significantly lower correlation with that of their barley homologs than do the 3' probesets. This can also be seen in the distribution of the expression values themselves in Panel C. In general, expression values as measured by the 5' probesets tends to be much lower than those of their barley counterparts (solid line), while the signal from the corresponding 3' probesets shows a distribution roughly similar to that of the barley homologs.

Individual 3' and 5' probesets can be inferred to correspond to the same gene if they both show strong sequence similarity to a barley sequence but at the same time do not have a significant similarity with each other. Indeed, of the approximately 5,300 consensus sequences from the 5' set homologous to a barley sequence, 78% can be associated to a wheat 3' sequence in this way. A subset of these sequences is contained in the class of homologous clusters shown in Figure 6A. There are 1,590 clusters of this type in our dataset.

The distribution of correlations of the expression profiles from this set of 3' and 5' wheat sequences is shown in Figure 6B. The typical correlation between the 3' wheat and barley profile is quite high (mean = 0.57), while the correlation between the 5' wheat and barley profiles tends to be considerably lower (mean = 0.32). It appears from this that while some 5' wheat probesets might very well give a reliable signal, on average the reliability is significantly lower than that of the 3' wheat probesets. Additional evidence to support this conclusion is shown in Figure 6C where the distribution in actual expression levels for both the wheat 3' and wheat 5' probesets is shown along with the expression levels in barley. It appears from this plot that the signal from the 5' probesets is consistently substantially lower than the signal from the barley probesets. Finally, a cursory inspection of profiles for any homologous cluster using the *WebComparator * shows that probesets from the 5' set often don't show any significant hybridization. We conclude that one needs to interpret the signal from these probesets with great care and for this reason we shall not include them in our comparative analyses.

### Conservation and divergence of gene function in wheat and barley

**Figure 7:**
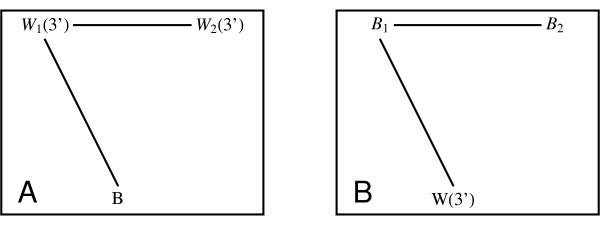
**Homologous clusters exhibiting greater intra-species than inter-species sequence similarities**. These types of clusters are indicative of a gene duplication having occurred in wheat (Panel A) or barley (Panel B). The absence of a significant Blast hit between W_2 _and B (or B_2 _and W) is consistent with subsequent sequence drift of one member of the duplicated pair.

Gene and protein expression studies indicate that, in general, sequence divergence after duplication events is associated with a divergence of functionality in the resulting paralogs, presumably because of reduced selection pressure after duplication [46,47]. In fact, it is widely believed that gene duplication – either individually or as part of genome-scale duplication events – is crucial for providing the resource for the subsequent evolution of genes with new functions [48,49]. Genome-wide studies of this sequence divergence have mostly been undertaken in sequenced organisms separated by reasonably large evolutionary distances. The wheat and barley tissues series permit such a study in these more closely related grass species. Consider homologous clusters of the type presented in Figure 7. The type of cluster shown in Figure 7A is consistent with a gene duplication event in wheat, with subsequent sequence divergence of sequence W_2_. Similarly, Figure 7B is consistent with a gene duplication in barley, with subsequent sequence divergence of sequence B_2_. The "high quality" dataset contains 24 and 94 examples of this type of cluster, respectively.

A measure of functional divergence, on the other hand, is once more provided by the correlation between wheat and barley expression profiles. For the cluster shown in Figure 7A, if the correlation between the profile for the barley sequence B and the wheat sequence W_1 _(*C(B, W*_1_)) is similar to the correlation between B and W_2_(*C(B, W*_2_)), one would conclude that there is little relation between functional and sequence divergence. On the other hand, *C*(*B*, *W*_2_) <*C*(*B*, *W*_1_) would indicate a possible relationship. The results of these comparisons are presented in Table 2. The average correlation between the profiles for B and W_1 _is higher than that for B and W_2_, and similarly the profiles for W and B_1 _are on average more correlated than those for W and B_2_. While the sample sizes are small, the P-values associated with a Wilcoxon signed-rank test indicate that these differences are likely to be significant. In short, wheat and barley apparently exhibit a correlation between divergence of gene function and gene sequence akin to that found in less closely related species.

**Table 2:** Expression profile conservation versus sequence conservation.

Gene pair	<C>	N	P-Value
B – W_1_	0.33		
		24	0.012
B – W_2_	0.15		

W – B_1_	0.51		
		94	0.087
W – B_2_	0.43		

The correlation of gene expression profiles in wheat and barley increases with similarity of gene sequence. Gene pairs refer to those shown in Figure 7A (top two rows) and 7B (bottom two rows). <C> refers to the average Pearson correlation for the expression profiles of these gene pairs. N is the sample size and the P-value is calculated using the Wilcoxon signed-rank test.

### Dosage constraints in wheat and barley

While gene duplication provides opportunities for the evolution of new gene function, it has also been argued that selective pressure can maintain original function after a duplication event. This might occur if the gene codes for part of a protein complex [50], thus imposing strong stoichiometric constraints, or if 'buffering' of crucial functions [51] is required. In addition, studies in yeast [52] and Paramecium [53] indicate that dosage constraints may account for the inhibition of divergence of duplicated genes. These authors found that, at least in these two species, duplicate copies of genes are more likely to be retained if the expression level is high than if it is low, presumably in order to maintain high transcript levels.

**Figure 8:**
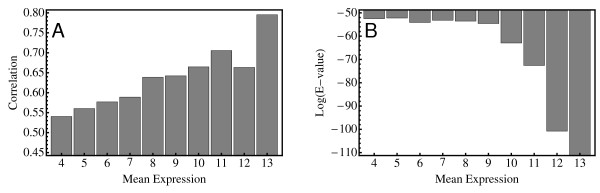
**Dosage constraints on gene evolution**. In Panel A, the correlation between expression profiles of wheat and barley genes linked by reciprocal Blast hits in the homologous clusters is plotted against the overall mean expression level for these gene pairs. The profiles exhibit higher correlation if the actual expression levels themselves are high, suggesting that gene function tends to be more conserved for highly expressed genes. In Panel B, the mean expression is plotted against sequence similarity (i.e. E-values of the Blast hits), indicating that there is an analogous correlation between the level of expression and sequence similarity.

We have compared the wheat and barley transcriptomes to see if such a correlation between maintenance of gene function and expression level persists in these two species. Again, we use conservation of the expression profiles (i.e. the correlation) between wheat and barley genes as an indirect measure of conservation of function. The results, using all wheat and barley probesets from the "high quality" set linked by a reciprocal Blast hit, are shown in Figure 8A. Genes that are expressed at a higher level tend to be genes which have more correlated expression profiles. In order to make sure that this is not an artefact of noise washing out underlying correlations at low expression levels we confirmed that the trends shown in Figure 8A persist even if genes with low expression levels (e.g. less than 7) in any tissue are left out of the analysis. Finally, one sees that the same correlation persists if one uses sequence similarity instead of profile similarity as a measure for conservation of gene function (Figure 8B). We conclude, therefore, that for wheat and barley the maintenance of high expression levels is a significant driver in maintaining gene function.

## Discussion

### Gene expression in wheat and barley is highly correlated

Our results (Figures 2 and 3) indicate highly correlated gene expression for the subset of barley and wheat genes in the 'high-quality set' that belong to homologous clusters with exactly one member from each species. While this restriction makes ortholog identification relatively unambiguous, it also leads to a biased sample: by construction, this selection is enriched for genes that are not members of gene families and/or genes where the sequence differences between homoeologs are small (or only one of the homoeologs is functional). It is quite possible, even plausible, that the average correlation of a broader gene set that includes members of gene families may differ from the value 0.66 observed in our more restricted set. Indeed, if the presence of gene family members is taken to indicate increased redundancy, one might well expect that the average correlation of expression levels might reduce. While, given the lack of reliable sequence information, it is not feasible to test this conjecture at present, it is interesting to note that our restricted sample of 1875 sequence pairs exhibits, on average, higher expression levels than the complete "high-quality set": expression levels in barley appear increased marginally by 37%, while in wheat they are roughly doubled.

### Conservation and divergence of gene function in wheat and barley

The evolution of new functional roles for duplicated genes can depend on a number of mechanisms. Most directly, accumulation of sequence changes in coding regions can lead to changes in protein structure, either by substitution of amino acids or through the evolution of new splice variants. This type of sequence divergence after gene duplication is well documented [47]. Numerous studies have observed the expected decreased selective pressure on sequence conservation after duplication through comparisons of synonymous and non-synonymous nucleotide substitutions [54]. A second mechanism is provided through alteration of gene regulation imparted through mutation in *cis*-acting elements and/or alterations of *trans*-acting factors. This alters a gene's expression repertoire even in the absence of changes in its sequence. The relationship between these two mechanisms also sheds light on the importance of selective pressure in the evolutionary process; random drift under a neutral model would result in unrelated expression and sequence changes, while strong selective pressure would be reflected by a positive correlation.

The results in Table 2 provide an indication that gene activity patterns across tissues and accumulation of sequence changes after gene duplication are positively correlated in wheat and barley, as one would expect in the presence of selective pressure [55]. This finding is in agreement with some of the studies in model species such as yeast [56], *C. elegans *[57], *Drosophila *[58] and primates [59]. However, these results are by no means universal and in other studies no clear correlation is observed [60]. For example, no [61] or weak [16] correlation was observed in two studies using the model plant *Arabidopsis*. It is likely that the lack of agreement reflects differences in approach; for example, in some studies sequence divergence is measured on the protein level, in others it is quantified through the rate of non-synonymous substitutions and others through the rate of synonymous substitutions. In some studies tandem duplications are separated out from ancient segmental duplications, while in others (including ours) they are not. Expression divergence, on the other hand, will clearly depend on the number and types of different temporal, spatial and environmental conditions which are probed.

We believe that at present the results shown in Table 2 should be seen as indicative only. Gene duplication and subsequent divergence is only one possible source for the apparently correlated divergence of sequence and expression. A second source for this correlation might be found in the generation of new alternative splice forms rather than new genes, with the original splice form maintaining its expression pattern but the new one diverging.

Furthermore, as opposed to the species mentioned above, wheat and barley do not have sequenced genomes and this naturally has an influence on the reliability of the available sequence and expression information. Here sequence divergence was assessed purely on the basis of the existence (for W_1 _and B_1 _in Figure 7) and absence (W_2 _and B_2_) of a Blast hit of sufficient quality. This is a cruder measure than a count of synonymous substitutions, and other effects such as a reduced sequence length of W_2 _and B_2 _due to incomplete EST information could easily impact on this. In addition, our sample size is quite small because it includes only those homologous graphs that we considered to be the most reliable, i.e. those containing the Blast hits of the type shown in Figure 7. Notwithstanding these qualifications, these latter issues would tend to reduce any underlying correlation, so the presence of these in Table 2 may actually be relatively robust. We also note that any remaining cross-hybridization between W_1 _and W_2 _(or B_1 _and B_2_), which we have sought to eliminate by considering only the "high-quality" dataset that doesn't contain "_x,s,a_at" probesets, would also tend to reduce any difference in the correlation signal. Improved knowledge of the sequence content of these two species will increase the reliability of the signal that we have observed.

A second effect apparent in Table 2 should be treated with even more caution. The expression profiles of duplicated wheat genes appear to be considerably less similar to that of their barley ortholog (correlation coefficients ~0.33 & 0.15) than duplicated barley genes and their wheat ortholog (correlation coefficients ~0.51 & 0.43). These correlations can be compared to those 1,875 sequence pairs discussed earlier where there is no evidence of gene and/or splice form duplication (correlation ~0.66). While it might be tempting to conclude that expression patterns in wheat evolve faster than their counterparts in barley, this signal is sensitive to any asymmetry in design of the two GeneChips. In particular, as discussed in [26] and at , the EST clustering procedures used in the construction of the Barley1 and Wheat Genechips was rather different, particularly in their treatment of potential splice-variants. It could well be these differences has led to differing proportions of probesets on the two GeneChips designed to individual splice forms.

Because both alternative splice forms as well as gene duplications potentially contribute to the asymmetries in Table 2, a differing admixture of the two could easily be responsible for this asymmetry between the species.

### Highly expressed genes show correlated expression in wheat and barley

Numerous studies have shown that genes transcribed at high levels tend to evolve more slowly than genes transcribed at low levels [62-64]. As already mentioned above, the "rate of evolution" in this type of study is quantified by sequence divergence, either on the DNA level by counting synonymous and/or non-synonymous substitutions or on the protein level by counting amino acid changes. The results shown in Figure 8B show that this correlation between sequence conservation and mean expression level can clearly be seen in the wheat and barley transcriptomes, even though we use the comparatively rough measure of a Blast hit E-value to quantify sequence similarity.

### Systematic shifts in expression levels and platform dependant biases

While differential hybridization signal (i.e. fluorescence) intensities in a select number of tissues are highly likely to be indicative of true biological differences between wheat and barley, great care must be taken in interpreting overall shifts of fluorescence levels in the same way. The RNA extractions for these experiments were performed in two different laboratories and, of course, with different species using GeneChips with two different design philosophies. RNA hybridization to the GeneChips, on the other hand, was performed at the same facility in a virtually identical manner. Even issues such as the poor hybridization efficiency of the large number of wheat 5' probesets are likely to have an impact on the relative normalization. Without an absolute standard, a rigorous relative calibration of the two datasets therefore seems very difficult, if not impossible. Our approach to this issue was two-fold: firstly, while one might not be able to control the overall normalization uncertainty one can attempt to estimate its importance and secondly, as described, we performed additional QPCR measurements in an attempt to verify interesting outcomes.

An estimate of the overall normalization uncertainty is provided by the data shown in Figure 4A. For these data, the average difference in (logarithmic) fluorescence levels is around 0.8, indicating that on average the fluorescence levels for the barley data are about 75% higher than the corresponding levels for the wheat data. Under the assumption that the true average expression level in the two species is similar one would conclude that overall normalization issues may account for a factor of around 2 between the datasets. The differences, often 10-fold or more, of the observed fluorescence levels shown in Figures 4C and 5 makes this an unlikely explanation.

For those probesets where there is a systematic decrease in the observed fluorescence levels in barley as compared to wheat, the barley QPCR results do not confirm the barley microarray results. This indicates that for these probesets, at least, the difference has a technical rather than biological origin. An obvious possibility for a lack of signal is the possible presence of single feature polymorphisms (SFPs; see Ref. [28]) between the probes on the Barley1 array and the Morex mRNA being hybridized to it. If this is the case, the rough consistency of QPCR and microarray results for wheat, for those probesets with enhanced microarray fluorescence levels in barley, would indicate that the SFPs do not play as much of a role in the hybridization of Chinese Spring to the wheat GeneChip as they do for the hybridization of Morex to the Barley1 GeneChip.

There is corroborating evidence supporting this conjecture. The Barley1 GeneChip was designed using the ESTs collected from 84 libraries, originating from EST projects in Japan, Finland, Germany, Scotland, and the US, respectively. Five major and a few minor cultivars were used, representing the favorite from each project. In the end, the majority of these ESTs were from Barke (Germany) and, to a lesser extent, Morex (US) [26]; in total, only about 1 in 7 ESTs used to design Barley1 came from Morex [28]. The dominant cultivar in the EST collections used to design the wheat GeneChip, on the other hand, is Chinese Spring. One would, therefore, expect a greater prevalence of SFPs between Morex mRNA and the probes on the Barley1 GeneChip as compared to SFPs between Chinese Spring mRNA and the probes on the wheat GeneChip. We have compared the sequences in current publicly available EST collections against the probe sequences on the two GeneChips and find that this is indeed the case: from this comparison, we estimate that the probability of any mismatch between a 25mer barley probe and Morex sequence to be around 2.9% while for the wheat GeneChip the probability of a mismatch between a probe and a Chinese Spring sequence is around 1.5%. Assuming independence, this implies that in Figure 4A of the order of ten or so Barley1 GeneChip probesets may contain more than two SFPs when compared to Morex sequences, while for Chinese Spring the effect is negligible. In other words, SFPs between the two cultivars and their respective GeneChips may indeed be responsible, qualitatively and quantitatively, for the discrepancies between the QPCR and microarray results observed for barley but not wheat.

## Conclusion

We have performed a comparative study of gene expression in barley and hexaploid wheat, using 13 different tissues and developmental stages. The comparison has been achieved through the clustering of almost 84,000 wheat and barley sequences represented on the Affymetrix wheat and barley GeneChips into homologous clusters, with over 10,700 clusters containing more than one sequence. Detailed comparisons of expression profiles for all of these sequences have been made available at  and individually the two gene expression atlases can be explored further at , accession numbers BB3 and TA3.

We have established that on the whole there are strong similarities between expression patterns of homologous genes in the two species. This conclusion could only be reached, however, by first taking into account the differing designs of the two GeneChips. Among several confounding factors, the most significant is the presence of over 32,000 probesets on the wheat GeneChip not clearly anchored to the 3' end of gene sequences. The expression profiles obtained with these probesets and, particularly, the comparison to expression profiles of homologous barley sequences clearly shows that most lead to a significantly compromised signal. In this way, our comparative results provide a significant resource aiding the interpretation of the hybridization signal from individual probesets in future experiments employing the wheat GeneChip.

Our results indicate that the hybridization signal obtained from the wheat GeneChip generally does not differentiate between wheat homoeologs. Detailed study of homoeolog expression patterns across tissues awaits the construction of microarray platforms that specifically target regions of homoeolog sequence divergence and/or studies employing direct transcriptome sequencing.

Finally, we have used several high-quality subsets of our expression datasets to investigate some of the more prominent, but nevertheless comparatively small, systematic differences between the wheat and barley data. As is to be expected, we found that great care must be taken to distinguish genuine differences in the transcriptomes from artifactual differences due either to the dissimilar design of the GeneChips and/or the disparity in our current knowledge of the wheat and barley genomes. Examples of the latter include a systematic shift in absolute expression found in a significant number of wheat and barley putative orthologs. On the other hand, we also found a comparatively clear indication that highly expressed wheat and barley genes tend to be evolutionarily conserved, both in sequence as well as transcriptional activity. This observation for these two grasses is in agreement with results from previous studies of model species.

## Methods

### Experiment design

Wild-type wheat (*Triticum aestivum *L. cv. Chinese Spring) was grown in a temperature-controlled growth room with 16 h light (22°C) and 8 h dark (16°C) at approximately 80% humidity. Thirteen plant tissues were selected to represent the major stages of wheat development and to mirror the experiment of Ref. [27] for barley (cv. Morex). The number of plants harvested and the developmental stages selected are as described in [27], with the following exception: while in the latter samples were collected for three stages of caryopsis (namely, 5, 10 and 16 DAP), for wheat only caryopsis 3–5 DAP was used. Three independent biological samples (replicates) represented a tissue type.

RNA isolation and quality checking were performed as described in Ref. [27]. Labeling and hybridization to the Affymetrix wheat GeneChip was carried out at the Iowa State University GeneChip facility . Background subtraction and normalization for both experiments was performed using the RMA normalization procedure [65,66] and the three biological replicates were averaged. The data is expressed on a logarithmic scale (base 2), as usual.

### Data access

All detailed data and protocols from these experiments have been deposited in PLEXdb , a unified public resource for gene expression for plants and plant pathogens. Files are categorized under accession TA3 for the wheat gene atlas and BB3 for the barley gene atlas. TA3 has also been deposited at NCBI-GEO as accession GSE12508.

### The Wheat GeneChip

It is crucial to take into account the different design philosophies of the Affymetrix wheat and barley GeneChips when comparing the transcriptome data obtained with them. In order to maximize the reliability of results we impose rigorous constraints to arrive at a set of probesets that may be judged to be reliable. The design of the Barley1 GeneChip has already been discussed in detail in Ref. [26]. Additional information on both GeneChips can be found in the technical support section of . Here we briefly summarize the relevant details of the wheat GeneChip. This GeneChip contains, apart from a small number of reporters and controls, 61,115 probesets. All but 73 of these are made up of 11 25-mer perfect-match (and accompanying mismatch) probes. As is usual for these GeneChips, there are a number of probesets where one or more probes are known to cross-hybridize in one way or another (for details, see Appendix B of the "GeneChip Expression analysis manual" available at ): their names are suffixed by "_s_at" (2617 probesets), "_x_at" (6766 probesets) and "_a_at" (3321 probesets). Because of the danger of unwanted cross-hybridization complicating ortholog identification across the two species, as well as the fact that in any case these probesets are often provided in addition to uniquely hybridizing probesets, we do not include them in the comparative analysis carried out in this paper. For the same reason, the results presented here only make use of those probesets from the Barley1 GeneChip for which ESTs could be assembled into a contig. Singleton ESTs tend to have shorter sequence, increasing the chance that confusion arises when trying to match them to a particular sequence present on the other GeneChip. Finally, in our comparative analysis we also disregard the 10,643 probesets marked with the suffix ".A1" because they are predominantly of the wrong orientation.

Furthermore, the wheat GeneChip includes a considerable number of probesets not clearly anchored to the 3' end (32,578 out of 61,115; Close and Davies, personal communication). These form part of the so-called "prune" set in Affymetrix's design pipeline and are usually used for checking probes for potential cross hybridization Throughout this paper we refer to these as "5' sequences". This type of sequence was not included on the Barley1 GeneChip because, while they may be useful for gene discovery, their hybridization efficiency is unreliable. Unless explicitly indicated otherwise, we do not consider them in our comparative analysis.

Finally, as discussed below, an additional quality control on the GeneChip sequences was obtained by demanding that the relative orientation of the consensus sequences on the barley and wheat GeneChips should be the same. Our comparative analysis leaves out sequences with opposite or inconsistent orientation on the two chips.

After all these cuts, 13,822 wheat probesets and 12,549 barley probesets remained and it was this set that we used. We stress, however, that expression results from *all *probesets have been included in the data contained in the *WebComparator *.

### Homolog identification on the Wheat and Barley GeneChips

We identified putative wheat and barley homologs using the following approach

1) After constructing non-redundant sets of consensus and exemplar sequences for the wheat and barley GeneChips, respectively, we performed all possible wheat-barley, barley-wheat, wheat-wheat and barley-barley sequence comparisons using NCBI's gapped Blastn [67] algorithm. The intra-species comparisons were performed in order to avoid, as much as possible, issues associated with the incomplete representation of the wheat and barley genomes on the two GeneChips.

2) A directed graph was constructed from the results of these sequence comparisons, with the nodes consisting of the non-redundant sequences. A directed edge starting at node *i *(being a sequence from genome *I*) and ending at node *j *(a sequence from genome *J*) was defined to exist if a) node *j *was the best Blast hit to node *i *when sequence *i *was compared to genome *J *and this Blast hit had an E-value better than the cut-off C = 10^-50^, or b) if the Blast hit had an E-value within a tolerance T = 10^-5 ^of that of the best Blast hit (if the best Blast hit had an E-value of 0 then this limit was taken to be within 10^-5 ^of machine precision instead). Note that keeping Blast hits which are close to the best Blast hit is useful if several homoeologs with near-identical sequence are present and/or if probesets have been tiled to both the 3' and 5' end of the same sequence, as was done for the wheat GeneChip.

3) Finally, the resulting graph was decomposed into connected sub-graphs (termed "homology graphs"), with those sequences contained within a sub-graph defining a putative "homologous cluster".

The results are quite insensitive to the choices for C and T; the precise value of C tends to be immaterial because either *I *= *J *(i.e. an intra-species comparison), in which case the best Blast hit naturally almost always links the sequence back to itself with an E-value of 0, or – if I ≠ J (an inter-species comparison) – the general similarity between wheat and barley sequences ensures that if a homolog is present at all it tends to have a similarity very much better than E ~ 10^-50^. The precise value of T, on the other hand, is not critical for a similar reason; most Blast hits are found to be either very close to the best Blast hit (usually with an E-value within a factor of 100 or so of the best E-value) or considerably further removed. In other words, while we have not attempted to distinguish homoeologs, paralogs and orthologs (only a phylogenetic treatment can do this), by using the above approach the detection of homologs in general is relatively unambiguous.

Typically, the homology graphs are rather small: only 105 out of a total of 10,708 non-trivial homology graphs contain more than 10 vertices. A much larger number of these graphs, just over 40,000, are found to be 'trivial' in the sense that they contain only 1 node, i.e. for these sequences, the Blast searches did not result in a significant hit to any other sequences. This should not be interpreted to mean that there are large numbers of genes in wheat and barley having no counterpart in the other species. Rather, inspection of the trivial graphs shows that about 57% of them correspond to 5' wheat sequences (presumably not having a significant overlap with the typically longer barley sequences) and slightly less than 10% correspond to short barley ESTs rather than longer contigs. It is to be expected that most of the remaining 13,000 sequences or so are unmatched because the two GeneChips do not represent the entire complement of genes from the two species. In principle, the number of trivial graphs could be reduced by increasing C considerably; however, we did not do so in order not to increase the number of false positive associations.

### Quantitative RT-PCR (QPCR) verifications

cDNA was synthesized from the same RNA that was hybridized to the wheat and barley GeneChips for 11 of the 13 tissues (excluding anthers and pistils). While three independent RNA samples were used in the microarray experiment, the QPCR cDNA was prepared for only one of the three RNA samples. Results from this sample were compared to the microarray results from the same sample. Templates of 5 μg total RNA for barley and 0.5 μg total RNA for wheat were used for the cDNA synthesis reaction with Superscript III RNAse H-Reverse Transcriptase (Invitrogen, Australia) according to the manufacturer's protocol.

Four control genes were assessed (actin, GAPdH, EFA and cyclophilin). The primers for the barley control genes are described in Ref. [68], while the wheat primers are listed in Table 3. The selection of barley and wheat probesets used for the comparison of microarray and QPCR results was drawn from the boxed region indicated in Figure 4A and is summarized in Table 4. Three groups of sequences were examined: two of these involved sequences (mostly sequence pairs) giving rise to intensity profiles similar to those shown in Figure 4C, with either wheat or barley having a higher overall intensity, while the third group consisted of sequence pairs whose fluorescence intensity profiles did not show significant intensity differences in the microarray experiments. These were either sequence pairs that did not show significant intensity differences in the microarray experiments or individual sequences used as internal controls for the QPCR experiments. Primer sequences designed from the corresponding consensus sequences are also shown in Table 4. Normalization factors were calculated from the three best control genes as described by Vandesompele et al. [69]. The QPCR was carried out as described by Crismani et al. [70].

**Table 3:** Q-PCR primer pairs used for the wheat control gene.

**Gene**	**Forward primer**	**Reverse primer**	**Product size (bp)**
ELF1	CAGATTGGCAACGGCTACG	CGGACAGCAAAACGACCAAG	227
GAPDH	TTCAACATCATTCCAAGCAGCA	CGTAACCCAAAATGCCCTTG	220
Cyclophilin	CAAGCCGCTGCACTACAAGG	AGGGGACGGTGCAGATGAA	227
Actin	GACAATGGAACCGGAATGGTC	GTGTGATGCCAGATTTTCTCCAT	236

Primer sequence details for the control genes used in the Q-PCR verification of selected wheat expression profiles and the size of the corresponding RNA products.

**Table 4:** Q-PCR primer pairs used to amplify selected barley and wheat transcripts.

**Probeset**	**Forward primer**	**Reverse primer**	**Product size (bp)**
**Barley1**			
Contig8230	TACATGCTCTTGTTTGGTGCTACTG	AAGGTAAGTAGGCAGCAGTGAAGGT	204
Contig6943	GGGGAAATCCCAGGTCGTCGAT	GGCTTGCTGCTAGGGTTTTCAG	266
Contig7925	CGAACCGTAGAATGTGTAAGGG	GGGAGGAAAGATACACGCTT	114
Contig3031	TTACTATGCTGGATATGGACAAGGG	TCTCATCTCATGTCTGGAAGACCC	190
Contig4668	CCCCCCACAAGTACCTGAAGA	CGTTGGCTTGCTTAGCTCTTCC	286
Contig7671	CTAAGCGACCTTGCATCTTTTGAC	AACGCTAGTGCTACTGGCAGGA	213
Contig20269	GAAGGCTCAGAAAGTTGCTGCTAT	GCAAAATCATTCACTGCTTCCAGAG	224
Contig5740	GAGGCTGTTCAGCAACTGGACTG	CAAGGATCCCAGCCACATACTG	227
Contig15148	GATCTCTTCGTGGTGGATCACATAC	GCTTGATGTCCTATGCTTTCCAA	221
Contig11660	ACCTCATCAACCTCTGCGGC	TTCCAGAGAACGGAGGCAGG	210
Contig14399	AGAAAGAGAGATTTTGAAGCTTGGC	AATCCATCGCCATGCCAACT	213
Contig15147	GGCGGGGCACTTTTGAGGACAT	CGAGCCTGCGACGGGTTATT	182
Contig2400	AAGCATGCCGCCATCCCGTT	CCCAACCTGACAACTCCACCTAGA	244
**Wheat**			
Ta.3039.1	ACGTCCATAACGATGGTCTTCATTG	GTAGTGGCCTCAGCATCACCATTGC	170
Ta.13729.1	TTTTCTACATGCTCTTGTTTGGTGC	AAAAGATCAACCCATGTGCTGCTCC	265
Ta.27013.1	CGAAGCGTGTATCTTTCCTC	CAGACACAAACGAAAATGAC	183
Ta.7602.1	AGCCCCCCACAAGTACCTGATGATG	GTCGTCATCCTCGTCACCATCTTCC	201
Ta.27369.1	TTACTATGCTGGATATGGACAAGGC	TGCTACAACATTAGCCTTGACAGTG	230
Ta.9536.1	GCCCTAAACGACCTTGCATCTTTTG	AAACTGAAGCACTAACCTACGACGC	236
Ta.968.1	ATGTGCTGCGTCGTCAGATACATAG	TACCCTCCTCGACTTCCTTGTGATC	204
Ta.4425.1	GATGCCATCAGATCCTCCAATT	GCCACTCCGTTGTGTCATAATATGG	235
Ta.27038.1	CGAAGCGTGTATCTTTCCTC	CAGACACAAACGAAAATGAC	151
Ta.7256.1	CATCTCATGGTACCTGACTGTCGA	GCAACAGACTGCCACCAGCA	264
TaAffx.46790.1	TCATGTCAGTTTATTGCAAGG	CAGTGACACTATAACAATACAGTTCT	240
TaAffx.128707.1	GAAAAGGTTGTAGTTCAGAAGG	TTGCTCTGGACTACTGTCTTC	259

Primer sequence details used in the Q-PCR verification of selected barley and wheat expression profiles and the size of the corresponding RNA products.

## Authors' contributions

AWS conceived and performed the analysis and drafted the manuscript. TS and GM grew the plants and extracted the mRNA. RAC and RPW organized and carried out the microarray hybridizations. EK created the *WebComparator *software application. BL performed the QPCR verifications. GJM, AD, RW, RPW and PL conceived, organized and obtained the funding for the collaborations behind wheat and barley tissues series. UB participated in the analysis and QPCR verification and together with RW participated in drafting the manuscript. All authors reviewed and edited the manuscript.

## References

[B1] Feuillet C, Langridge P, Waugh R (2008). Cereal breeding takes a walk on the wild side. Trends Genet.

[B2] Zohary D, Hopf M (2001). Domestication of plants in the Old World.

[B3] Devos KM, Gale MD (1997). Comparative genetics in the grasses. Plant Mol Biol.

[B4] Ramakrishna W, Dubcovsky J, Park YJ, Busso C, Emberton J, SanMiguel P, Bennetzen JL (2002). Different types and rates of genome evolution detected by comparative sequence analysis of orthologous segments from four cereal genomes. Genetics.

[B5] Islam AKMR, Shepherd KW, Sparrow DHB (1981). Isolation and characterization of euplasmic wheat-barley chromosome addition lines. Heredity.

[B6] Adams KL, Wendel JF (2005). Polyploidy and genome evolution in plants. Curr Opin Plant Biol.

[B7] Levy AA, Feldman M (2004). Genetic and epigenetic reprogramming of the wheat genome upon allopolyploidization. Biol J Linn Soc.

[B8] Kashkush K, Feldman M, Levy AA (2003). Transcriptional activation of retrotransposons alters the expression of adjacent genes in wheat. Nat Genet.

[B9] He P, Friebe B, Gill B, Zhou J-M (2003). Allopolyploidy alters gene expression in the highly stable hexaploid wheat. Plant Mol Biol.

[B10] Paterson A, Bowers JE, Chapman BA (2004). Ancient polyploidization predating divergence of the cereals, and its consequences for comparative genomics. Proc Natl Acad Sci USA.

[B11] Adams KL, Cronn R, Percifield R, Wendel JF (2003). Genes duplicated by polyploidy show unequal contributions to the transcriptome and organ-specific reciprocal silencing. Proc Natl Acad Sci USA.

[B12] Feldman M, Levy AA (2005). Allopolyploidy-a shaping force in the evolution of wheat genomes. Cytogenet Genome Res.

[B13] Bottley A, Xia GM, Koebner RMD (2006). Homoeologous gene silencing in hexaploid wheat. Plant J.

[B14] Bottley A, Koebner RMD (2008). Variation for homoeologous gene silencing in hexaploid wheat. Plant J.

[B15] Kashkush K, Feldman M, Levy AA (2002). Gene loss, silencing, and activation in a newly synthesized wheat allotetraploid. Genetics.

[B16] Blanc G, Wolfe KH (2004). Functional divergence of duplicated genes formed by polyploidy during Arabidopsis evolution. Plant Cell.

[B17] Ma L, Chen C, Liu X, Jiao Y, Su N, Li L, Wang X, Cao M, Sun N, Zhang X (2005). A microarray analysis of the rice transcriptome and its comparison to Arabidopsis. Genome Res.

[B18] Flagel L, Udall J, Nettleton D, Wendel J (2008). Duplicate gene expression in allopolyploid Gossypium reveals two temporally distinct phases of expression evolution. BMC Biology.

[B19] Quesada T, Li Z, Dervinis C, Li Y, Bocock PN, Tuskan GA, Casella G, Davis JM, Kirst M (2008). Comparative analysis of the transcriptomes of *Populus trichocarpa *and *Arabidopsis thaliana *suggests extensive evolution of gene expression regulation in angiosperms. New Phytologist.

[B20] Krom N, Ramakrishna W (2008). Comparative analysis of divergent and convergent gene pairs and their expression patterns in rice, Arabidopsis, and *Populus*. Plant Physiol.

[B21] Mangelsen E, Kilian J, Berendzen KW, Kolukisaoglu ÜH, Harter K, Jansson C, Wanke D (2008). Phylogenetic and comparative gene expression analysis of barley (Hordeum vulgare) WRKY transcription factor family reveals putatively retained functions between monocots and dicots. BMC Genomics.

[B22] Horvarth DP, Schaffer R, West M, Wisman E (2003). Arabidopsis microarrays identify conserved and differently expressed genes involved in shoot growth and development from distantly related plant species. Plant J.

[B23] Becher M, Talke IN, Krall L, Krämer U (2004). Cross-species microarray transcript profiling reveals high constitutive expression of metal homeostasis genes in shoots of the zinc hyperaccumulator Arabidopsis halleri. Plant J.

[B24] Ueda A, Kathiresan A, Bennet J, Takabe T (2006). Comparative transcriptome analyses of barley and rice under salt stress. Theor Appl Genet.

[B25] Taji T, Seki M, Satou M, Sakurai T, Kobayashi M, Ishiyama K, Narusaka Y, Narusaka M, Zhu J-K, Shinozaki K (2004). Comparative genomics in salt tolerance between Arabidopsis and Arabidopsis-related halophyte salt cress using Arabidopsis microarray. Plant Physiol.

[B26] Close TJ, Wanamaker SI, Caldo RA, Turner SM, Ashlock DA, Dickerson JA, Wing RA, Muehlbauer GJ, Kleinhofs A, Wise RP (2004). A new resource for cereal genomics: 22K barley GeneChip comes of age. Plant Physiol.

[B27] Druka A, Muehlbauer G, Druka I, Caldo R, Baumann U, Rostoks N, Schreiber A, Wise R, Close T, Kleinhofs A, Graner A, Schulman A, Langridge P, Sato K, Hayes P, McNicol J, Marshall D, Waugh R (2006). An atlas of gene expression from seed to seed through barley development. Funct Integr Genomics.

[B28] Rostoks J, Borevitz JO, Hedley PE, Russell J, Mudie S, Morris J, Cardle L, Marshall DF, Waugh R (2005). Single-feature polymorphism discovery in the barley transcriptome. Genome Biol.

[B29] Potokina E, Druka A, Luo Z, Wise R, Waugh R, Kearsey M (2008). Gene expression quantitative trait locus analysis of 16 000 barley genes reveals a complex pattern of genome-wide transcriptional regulation. Plant J.

[B30] Potokina E, Druka A, Luo Z, Moscou M, Wise R, Waugh R, Kearsey M (2008). Tissue-dependent limited pleiotropy affects gene expression in barley. Plant J.

[B31] Poole R, Barker G, Wilson ID, Coghill JA, Edwards KJ (2007). Measuring global gene expression in polyploidy; a cautionary note from allohexaploid wheat. Funct Integr Genomics.

[B32] Luo ZW, Potokina E, Druka A, Wise R, Waugh R, Kearsey MJ (2007). SFP genotyping from Affymetrix arrays is robust but largely detects *cis*-acting expression regulators. Genetics.

[B33] Mochida K, Yamazaki Y, Ogihara Y (2003). Discrimination of homoeologous gene expression in hexaploid wheat by SNP analysis of contigs grouped from a large number of expressed sequences tags. Mol Genet Genomics.

[B34] Tatusov RL, Galperin MY, Natale DA, Koonin EV (2000). The COG database: a tool for genome-scale analysis of protein functions and evolution. Nucl Acids Res.

[B35] Tatusov RL, Natale DA, Garkavtsev IV, Tatusova TA, Shankavaram UT, Rao BS, Kiryutin B, Galperin MY, Fedorova ND, Koonin EV (2001). The COG database: new developments in phylogenetic classification of proteins from complete genomes. Nucl Acids Res.

[B36] Tatusov RL, Fedorova ND, Jackson JD, Jacobs AR, Kiryutin B, Koonin EV, Krylov DM, Mazumder R, Mekhedov SL, Nikolskaya AN, Rao BS, Smirnov S, Sverdlov AV, Vasudevan S, Wolf YI, Yin JJ, Natale DA (2003). The COG database: an updated version includes eukaryotes. BMC Bioinformatics.

[B37] Lee Y, Sultana R, Pertea G, Cho J, Karamycheva S, Tsai J, Parvizi B, Cheung F, Antonescu V, White J, Holt I, Liang F, Quackenbush J (2002). Cross-referencing eukaryotic genomes: TIGR orthologous gene alignments (TOGA). Genome Res.

[B38] O'Brien KP, Remm M, Sonnhammer EL (2005). Inparanoid: a comprehensive database of eukaryotic orthologs. Nucleic Acids Res.

[B39] Tatusov RL, Mushegian AR, Bork P, Brown NP, Hayes WS, Borodovsky M, Rudd KE, Koonin EV (1996). Metabolism and evolution of *Haemophilus influenzae *deduced from a whole-genome comparison with *Escherichia coli*. Curr Biol.

[B40] Hirsh AE, Fraser HB (2001). Protein dispensability and rate of evolution. Nature.

[B41] Jordan IK, Rogozin IB, Wolf YI, Koonin EV (2002). Essential genes are more evolutionarily conserved than are nonessential genes in bacteria. Genome Res.

[B42] Wall DP, Fraser HB, Hirsh AE (2003). Detecting putative orthologs. Bioinformatics.

[B43] Tatusov RL, Koonin EV, Lipman DJ (1997). A genomic perspective on protein families. Science.

[B44] Remm M, Storm CEV, Sonnhammer ELL (2001). Automatic clustering of orthologs and in-paralogs from pairwise species comparisons. J Mol Biol.

[B45] Zhang P, Gu Z, Li WH (2003). Different evolutionary patterns between young duplicate genes in the human genome. Genome Biol.

[B46] Fulton DL, Li YY, Laird MR, Horsman BGS, Roche FM, Brinkman FSL (2006). Improving the specificity of high-throughput ortholog prediction. BMC Bioinformatics.

[B47] Lynch M, Conery JS (2000). The evolutionary fate and consequences of duplicate genes. Science.

[B48] Ohno S (1970). Evolution by gene duplication.

[B49] Taylor JS, Raes J (2004). Duplication and divergence: The evolution of new genes and old ideas. Ann Rev Genet.

[B50] Papp B, Pal C, Hurst LD (2003). Dosage sensitivity and the evolution of gene families in yeast. Nature.

[B51] Chapman BA, Bowers JE, Feltus FA, Paterson AH (2005). Buffering of crucial functions by paleologous duplicated genes may contribute cyclicality to angiosperm genome duplication. Proc Natl Acad Sci USA.

[B52] Seoighe C, Wolfe KH (1999). Yeast genome evolution in the post-genome era. Curr Opin Microbiol.

[B53] Aury J-M, Jaillon O, Duret L, Noel B, Jubin C (2006). Global trends of whole-genome duplications revealed by theciliate *Paramecium tetraurelia*. Nature.

[B54] Zhang J (2003). Evolution by gene duplication: an update. Trends Ecol Evol.

[B55] Li W-H, Yang J, Gu X (2005). Expression divergence between duplicate genes. Trends Genet.

[B56] Gu Z, Nicolae D, Lu HH-S, Li W-H (2002). Rapid divergence in expression between duplicate genes inferred from microarray data. Trends Genet.

[B57] Castillo-Davis CI, Hartl DL, Achaz G (2004). *cis*-regulatory and protein evolution in orthologous and duplicate genes. Genome Res.

[B58] Nuzhdin SV, Wayne ML, Harmon KL, McIntyre LM (2004). Common pattern of evolution of gene expression level and protein sequence in *Drosophila*. Mol Biol Evol.

[B59] Makova KD, Li W-H (2003). Divergence in the spatial pattern of gene expression between human duplicate genes. Genome Res.

[B60] Wagner A (2000). Decoupled evolution of coding regions and mRNA expression patterns after gene duplication: implications for the neutralist-selectionist debate. Proc Natl Acad Sci USA.

[B61] Haberer G, Hindemitt T, Meyers BC, Mayer KFX (2004). Transcriptional similarities, dissimilarities and conservation of *cis*-elements in duplicated genes of Arabidopsis. Plant Physiol.

[B62] Pal C, Papp B, Hurst LD (2001). Highly expressed genes in yeast evolve slowly. Genetics.

[B63] Drummond DA, Bloom JD, Adami C, Wilke CO, Arnold FH (2005). Why highly expressed proteins evolve slowly. Proc Natl Acad Sci USA.

[B64] Choi JK, Kim SC, Seo J, Kim S, Bhak J (2007). Impact of transcriptional properties on essentiality and evolutionary rate. Genetics.

[B65] Irizarry RA, Hobbs B, Collins F, Beazer-Barclay YD, Antonellis KJ, Scherf U, Speed TP (2003). Exploration, normalization and summaries of high density oligonucleotide array probe level data. Biostatistics.

[B66] Bolstad BM, Irizarry RA, Astrand M, Speed TP (2003). A comparison of normalization methods for high density oligonucleotide array data based on bias and variance. Bioinformatics.

[B67] Altschul S, Madden T, Schaffer A, Zhang J, Zhang Z, Miller W, Lipman D (1997). Gapped BLAST and PSI-BLAST: a new generation of protein database search programs. Nucleic Acids Res.

[B68] Lunde C, Baumann U, Shirley NJ, Drew DP, Fincher GB (2006). Gene structure and expression pattern analysis of three *monodehydroascorbate reductase (Mdhar) *genes in *Physcomitrella patens*: Implications for the evolution of the MDHAR family in plants. Plant Mol Biol.

[B69] Vandesompele J, De Preter K, Pattyn F, Poppe B, Van Roy N, De Paepea A, Speleman F (2002). Accurate normalization of real-time quantitative RT-PCR data by geometric averaging of multiple internal control genes. Genome Biol.

[B70] Crismani W, Baumann U, Sutton T, Shirley N, Webster T, Spangenberg G, Langridge P (2006). Microarray expression analysis of meiosis and microsporogenesis in hexaploid bread wheat. BMC Genomics.

